# Volatile Emissions and Relative Attraction of the Fungal Symbionts of Tea Shot Hole Borer (Coleoptera: Curculionidae) †

**DOI:** 10.3390/biom12010097

**Published:** 2022-01-07

**Authors:** Paul E. Kendra, Nurhayat Tabanca, Luisa F. Cruz, Octavio Menocal, Elena Q. Schnell, Daniel Carrillo

**Affiliations:** 1 United States Department of Agriculture, Agricultural Research Service, Subtropical Horticulture Research Station, Miami, FL 33158, USA; nurhayat.tabanca@usda.gov (N.T.); elena.schnell@usda.gov (E.Q.S.); 2 Tropical Research and Education Center, University of Florida, Homestead, FL 33031, USA; luisafcruz@ufl.edu (L.F.C.); omenocal18@ufl.edu (O.M.); dancar@ufl.edu (D.C.)

**Keywords:** ambrosia beetles, chemical ecology, *Euwallacea perbrevis*, *Fusarium* dieback, invasive species, *p*-menth-2-en-1-ol, quercivorol, symbiosis

## Abstract

*Euwallacea perbrevis* is an ambrosia beetle that vectors fungal pathogens causing *Fusarium* dieback in Florida avocado trees. Current monitoring lures contain quercivorol, a fungus-produced volatile, but the exact attractant is unknown since lures contain a mixture of *p*-menth-2-en-1-ol isomers and both α- and β-phellandrene. This study used pure cultures of six symbiotic fungi isolated from *E. perbrevis* to document volatile emissions and determine the relative attraction of symbionts in binary choice assays. In a comparative test, headspace solid-phase microextraction followed by gas chromatography–mass spectroscopy was used to identify and quantify emissions from 3-week-old cultures. In a temporal study, Super-Q collection followed by gas chromatography–flame ionization detection was used to measure *cis*- and *trans-p*-menth-2-en-1-ol emissions for three months. A total of 15 compounds were detected, with monoterpene hydrocarbons and oxygenated monoterpenoids predominating. Only *trans-p*-menth-2-en-1-ol was common to all six symbionts. Peak levels of both isomers were observed at day 7, then gradually declined over a 90 day period. In choice tests, avocado sawdust disks inoculated with *Fusarium* sp. nov. were the most attractive. This symbiont produced only two volatiles, *trans-p*-menth-2-en-1-ol and limonene. The combined results indicate that *trans-p*-menth-2-en-1-ol is the primary female attractant emitted from symbiotic fungi, but limonene may be a secondary attractant of *E. perbrevis*.

## 1. Introduction

Shot hole borers in the genus *Euwallacea* (Coleoptera: Curculionidae: Scolytinae) are Asian ambrosia beetles that comprise a cryptic species complex newly invasive in the continental United States and in other countries. Species established in the USA include the tea shot hole borer (*E. perbrevis* Schedl) in Florida, and the polyphagous shot hole borer (*E. fornicatus* Eichhoff) and Kuroshio shot hole borer (*E. kuroshio* Gomez and Hulcr) in California [1]. Typical of ambrosia beetles, females are wood borers that colonize host trees in which they cultivate symbiotic fungi as a nutritional source for larvae and adults [2]. The exotic fungi vectored by *Euwallacea* spp. are phytopathogenic and induce *Fusarium* dieback, a destructive vascular disease of avocado, woody ornamentals, and numerous native American trees [3,4].

*Euwallacea perbrevis* in Florida (referred to as *E*. nr. *fornicatus* in previous published reports [4,5] prior to the taxonomic revision by Smith et al. [1]) escalated in pest status in 2016 when extensive infestations were detected throughout the commercial avocado production area of Miami-Dade County [4]. Although tea [*Camellia sinensis* (L.) Kuntz] is commonly attacked in Asia [6], avocado (*Persea americana* Mill.) appears to be the preferred host in Florida [4,5], but colonization of other fruit trees and natives has been observed [7]. Avocado is an important agricultural commodity in south Florida, with a production value of $23.6 million for the 2019–2020 season [8]. The prevalence of *Fusarium* dieback presents additional challenges to Florida growers, who are already experiencing significant losses due to laurel wilt, a lethal vascular disease of the Lauraceae, including avocado. Laurel wilt is caused by *Raffaelea lauricola* T. C. Harr., Fraedrich and Aghayeva, the primary fungal symbiont of redbay ambrosia beetle, *Xyleborus glabratus* Eichhoff [9,10]; however, this symbiont has since transferred laterally to multiple species of ambrosia beetle in Florida, which now function as additional vectors [11,12].

Field monitoring of *E. perbrevis* populations was first achieved using lures containing quercivorol, a food-based attractant emitted by symbiotic fungi [13]. Lures were deployed in either Lindgren funnel traps or sticky panel traps [4,5,14]. Subsequent studies showed that the α-copaene lure developed for redbay ambrosia beetle [15] also improved captures of *E. perbrevis* when combined with quercivorol [5,16,17]. Analysis of the commercial quercivorol lure indicated that it contained four enantiomers of *p*-menth-2-en-1-ol as well as α- and β-phellandrene; the percent composition consisted of two *trans*-isomers: 48.12% (1*R*,4*S*)-*p*-menth-2-en-1-ol and 39.97% (1*S*,4*R*)-*p*-menth-2-en-1-ol, two *cis*-isomers: 4.95% (1*R*,4*R*)-*p*-menth-2-en-1-ol and 3.98% (1*S*,4*S*)-*p*-menth-2-en-1-ol, 1.57% β-phellandrene, and 1.42% α-phellandrene [16]. The common name ‘quercivorol’ refers only to (1*S*,4*R*)-*p*-menth-2-en-1-ol [18], which is not the predominant component in the lure, so it is not clear which enantiomer(s) is/are attractive to *E. perbrevis*. It has also not been determined which fungal symbionts emit volatiles attractive to the beetle, warranting further investigation of the chemical ecology underpinning the interaction of *E. perbrevis* and its fungal symbionts.

In this study, we (1) prepared pure laboratory cultures of six fungal symbionts isolated from *E. perbrevis*, (2) identified and quantified the chemical emissions from each symbiont using volatile collections by headspace solid-phase microextraction (HS-SPME) coupled with gas chromatography–mass spectroscopy (GC–MS), (3) documented the pattern of Super-Q collected emissions of *cis*- and *trans*-*p*-menth-2-en-1-ol for 90 days by gas chromatography–flame ionization detection (GC–FID), and confirmed by GC–MS, from a symbiont that emitted both isomers, and (4) conducted binary choice bioassays with female *E. perbrevis* to determine relative attraction to symbionts.

## 2. Materials and Methods

### 2.1. Fungal Cultures

Six *E. perbrevis* fungal associates—*Fusarium* sp. AF-6 (AF-6), *Fusarium* sp. AF-8 (AF-8), *Fusarium* sp. nov., *Graphium* sp., *Acremonium* sp., and *Acremonium murorum*—were obtained from stock cultures isolated and identified at the Tropical Fruit Entomology Laboratory, University of Florida, Tropical Research and Education Center (TREC), Homestead, FL, USA. Sequences of the strains used in this study were deposited into GenBank (AF-6: translation elongation factor 1-α (EF1-α)-MZ265351, DNA-directed RNA polymerase subunit 1 (RBP1)-MZ265354, second-largest subunit of RNA polymerase (RBP2)-MZ265357; AF-8: EF1-α-MZ265350, RBP1-MZ265353, RBP2-MZ265356; *Fusarium* sp. nov: EF1-α-MZ265349, RPB1-MZ265352, RPB2-MZ265355; *Graphium* sp.: 28S large ribosomal subunit (LSU)-MZ262759, 18S small ribosomal subunit (SSU)-MZ262756; *Acremonium* sp.: LSU-MZ262757, SSU-MZ262754; *A. murorum*: LSU-MZ262757, SSU-MZ262755). Fungal strains were cultured on 100 mm (diam) × 15 mm Petri dishes containing potato dextrose agar media (PDA) (BD, Franklin Lakes, NJ, USA) (Fisher Scientific, Waltham, MA, USA). For the bioassays, 100 µL of 1 × 10^6^ spores/mL suspensions of *Fusarium* sp. nov., AF-8 and *Graphium* sp. were individually plated on 47 mm (diam) Petri dishes containing avocado sawdust media [19]. PDA and sawdust media plates were incubated at 25 °C.

### 2.2. Test Chemicals

Chemical standards consisted of α-phellandrene #99-83-2, α-terpinene #99-86-5, *p*-cymene #99-87-6, limonene #5989-54-8, terpinen-4-ol #20126-76-5, and α-terpineol #98-55-5 (purchased from Sigma-Aldrich, St. Louis, MO, USA); β-elemene #515-13-9 and valencene #4630-07-3 (purchased from Fluka Chemical Co., Buchs, SG, Switzerland); and *cis*-*p*-menth-2-en-1-ol and *trans*-*p*-menth-2-en-1-ol mixture (commercial quercivorol lure, Product #3402, obtained from Synergy Semiochemicals Corp., Delta, BC, Canada).

### 2.3. Volatile Extraction

Mycelial cultures on PDA Petri dishes of *A. murorum*, *Acremonium* sp., AF-6, AF-8, *Fusarium* sp. nov. and *Graphium* sp. were sampled for volatile emissions when 24–25 days old. Collection of volatile compounds was performed using a SPME fiber coated with 50/30 μm divinylbenzene/carboxen on polydimethylsiloxane (DVB/CAR/PDMS). Each fungal culture was placed in a Pyrex cylindrical jar then sealed with plastic film and left at 25 °C for 30 min for equilibration of volatiles in the headspace. After equilibration, the film was pierced with the SPME needle, and the fiber exposed to the sample headspace for 1 h at 25 °C. After the extraction, the fiber was immediately inserted into the injector port of the GC–MS for desorption 2 min in the splitless mode.

### 2.4. Gas Chromatography–Mass Spectrometry Analysis

Volatile emissions were analyzed by GC–MS Agilent 7890B GC coupled with 5977B mass selective detector (Agilent Technologies, Santa Clara, CA, USA). A DB-5 column (30 m × 0.25 mm inner diameter with 0.25 μm film thickness) was used and GC oven temperature program was 60 °C for 1.3 min, rose at 3 °C/min up to 246 °C. The injector and detector temperatures were kept at 220 °C and 230 °C. Helium was used as a carrier gas at a flow rate of 1.3 mL/min. Mass spectra were recorded at 70 eV. Mass range was *m*/*z* 35 to 450 Da and the scan rate was 2.8 scans/s. Mass Hunter B.07.06 software (Agilent Technologies) was used for data acquisition and processing. Volatile compounds emitted by symbionts were identified by comparing retention times, retention indices (RI) calculated using the Van den Dool and Kratz [20] equation in relation to a homologous series of *n*-alkanes (C_9_–C_21_) and libraries MassFinder [21], Adams Library [22], Flavours and Fragrances of Natural and Synthetic Compounds 3 [23], and Wiley 12/NIST 2020 [24], and in-house library “SHRS Essential Oil Constituents-DB-5” which was built up from authentic standards and components of known essential oils [5,7,16]. Results expressed as the relative percentage of each compound peak area to the total GC–MS peak area. Compounds released from Petri plates without fungal culture and siloxanes in procedural blanks as background contamination produced by SPME fiber were subtracted. All sample preparations and analyses were independently performed in triplicate.

### 2.5. Collection and Quantification of p-Menth-2-en-1-ols

Volatile emissions of *cis*-*p*-menth-2-en-1-ol and *trans*-*p*-menth-2-en-1-ol from *Graphium* sp. cultures on PDA Petri plates (four replicates) were sampled over the course of three months every two or three days using Super-Q adsorbent (Analytical Research Systems, Gainesville, FL, USA). Cultures were stored for the duration of this study at 25 °C in an incubator (Shel lab, model SRI20PF, Sheldon Mfg. Inc., Cornelius, NC, USA). Super-Q volatile collections were conducted as described previously [16] but using a slightly modified version. Small oven bags (Reynolds, Richmond, VA, USA) were used for the collection of volatile emissions from each Petri plate, using two port holes to connect to the volatile collection system. Filtered air was introduced at 1 L/min through one port hole; a Super-Q trap collector attached to a vacuum line pulling at 1 L/min was set up on the other port hole (push-pull system). Collection of volatiles was timed for 15 min. Volatile emissions were eluted from the Super-Q adsorbent using 200 μL methylene chloride (#75-09-2, Sigma-Aldrich, St. Louis, MO, USA) and an internal standard equivalent to 5 μg of C_16_ (hexadecane #544-76-3, Sigma-Aldrich, St. Louis, MO, USA) was added to each sample prior to injection onto the GC analysis. 

For quantitative analysis, samples were analyzed using a 7890B Agilent GC with a flame ionization detector (FID) and a split-splitless injector. The column was an Agilent DB-5 30 m × 0.25 mm × 0.25 μm (Agilent Technologies, Santa Clara, CA, USA). Injector temperature was 220 °C; split ratio was 20:1. The carrier gas was helium with a total flow of 27.2 mL/min and a pressure of 14.4 psi. The oven temperature was programmed at 60 °C for 1.3 min increasing at 3 °C/min to reach a final temperature of 246 °C. The FID was set to 250 °C. Extracts were quantified by GC–FID and analyzed by GC–MS for comparison purposes only.

### 2.6. Experimental Insects

Mature *E. perbrevis* females were obtained from laboratory colonies maintained at TREC, using rearing methods described previously by Cruz et al. [25]. Briefly, *E. perbrevis* females were excavated from avocado logs collected from an orchard in Miami-Dade County, Florida, USA (25°31′31″ N, 80°29′7″ W). An avocado sawdust rearing medium was prepared and poured into 50 mL conical centrifuge tubes [19]. Females were surface-disinfested with 70% (*v*/*v*) ethanol for 5–7 s and individually placed into the tubes. Rearing tubes were capped with lids fitted with a 1 cm metallic screen opening for airflow and maintained at 25 °C and 75% relative humidity, with a 16:8 L:D photoperiod.

### 2.7. Laboratory Bioassays

Dual-choice bioassays were conducted in shallow plastic containers (21 cm long × 15 cm wide × 8 cm high) lined with white paper (Gordon Paper Co., Inc., Virginia Beach, VA, USA; BWKB18401000) to provide a rough surface upon which the beetles could walk, comparable to test arenas described previously [26]. Avocado sawdust disks inoculated with *Fusarium* sp. nov., AF-8 or *Graphium* sp. were placed on opposite sides of the container. A plastic vial containing six *E. perbrevis* females (≤7 days post-emergence) was placed at the center of each container. After a 5 min acclimation period, the vial was removed to allow the females to interact with the inoculated avocado sawdust disks. The container was then covered with a fitted lid with two screened ventilation holes and kept in a walk-in-rearing room at 25 °C in complete darkness. The number of beetles on, in or next to each avocado sawdust disk was recorded at one time point after 12 h. Beetles that did not choose a given treatment (not in, on or next to an avocado sawdust disk) were excluded from the analysis. Each combination of symbionts was replicated six times, reversing the position of the symbionts, and using new cohorts of beetles each time. 

### 2.8. Statistical Analysis

The data obtained from GC–MS were subjected to multivariate analysis. Principal component analysis (PCA) was performed using XLSTAT 2021 (Addinsoft, New York, NY, USA). Pearson correlation was adopted. A hierarchical cluster analysis (HCA) was performed using JMP Pro.16.0 software (SAS Institute Inc., Cary, NC, USA) using Ward’s clustering method. Regression analysis with exponential decay models was used to analyze emissions of *cis*- and *trans-p*-menth-2-en-1-ol from the *Graphium* sp. cultures; analysis by *t*-test was used to assess for differences in emissions of the two isomers at each sampling point (SigmaPlot 14.0, Systat Software Inc., San Jose, CA, USA). The results of the dual-choice bioassays were analyzed using a Kruskal–Wallis non-parametric test (SAS Institute Inc., Cary, NC, USA).

## 3. Results and Discussion

### 3.1. Volatile Emissions of Symbionts

**Figure 1:**
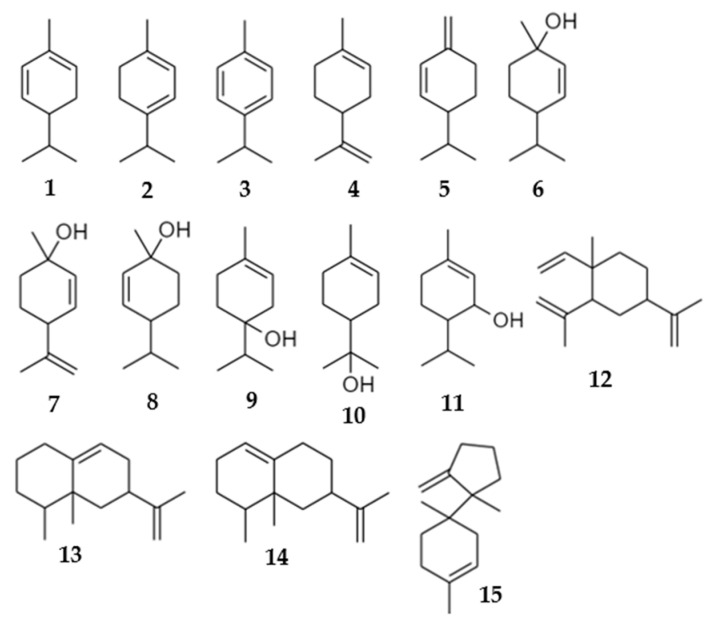
Structure of compounds presented in Table 1.

**Figure 2:**
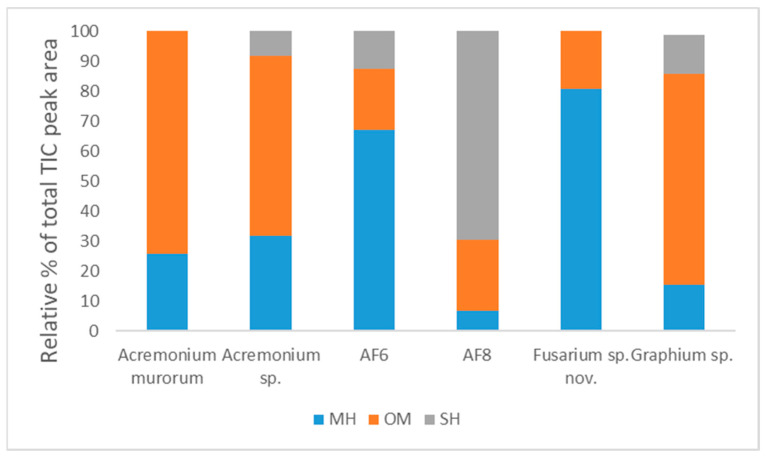
Percent composition of terpenoid classes in the fungal symbionts of *E. perbrevis*. MH—monoterpene hydrocarbons; OM—oxygenated monoterpenes; SH—sesquiterpene hydrocarbons.

SPME–GC–MS volatile compositions for individual fungal symbionts of *E. perbrevis* are presented as the peak area percentages in Table 1. A total of 15 compounds were detected from the emissions of the six symbionts, and the chemical structures of those compounds are illustrated in Figure 1. Notably, the monoterpene hydrocarbons and oxygenated monoterpenoids were the most abundant terpenoid classes emitted by the symbionts. Limonene and *trans-p*-menth-2-en-1-ol were the major constituents in the group of monoterpenoids in five symbionts, while aristolochene and valencene were higher in symbiont AF-8 than in *Acremonium* sp., AF-6 and *Graphium* sp. (Figure 2). Regarding the components present in the commercial quercivorol lure [16], *trans-p*-menth-2-en-1-ol was the only volatile detected across all six symbionts. *cis-p*-Menth-2-en-1-ol was detected in the emissions from *Graphium* sp. and both *Acremonium* spp. but was absent in the emissions from AF-6, AF-8, and *Fusarium* sp. nov. α-Phellandrene was detected in the emissions from *A. murorum* and *Graphium* sp., while β-phellandrene was emitted from cultures of *A. murorum*, AF-8, and *Graphium* sp.

**Figure 3:**
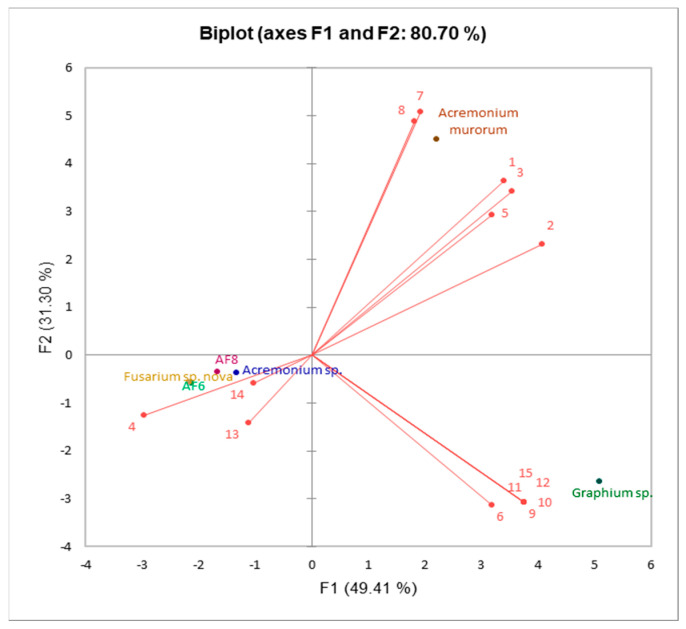
Principal component analysis (PCA) plot of the scores (volatile compounds emitted by symbionts) loading by dotes and loadings (symbionts) indicated by lines based on the first and second principal components. Numbers represent the compounds **1** to **15** in Table 1.

The results of principal component analysis (PCA) are indicated by the principal components (PC) score and loading plots (PCA-Biplot). The PCA of volatile components yielded three PCs with eigenvalues ≥ 1, accounting for 97.01% of the total variance across the entire dataset. The first, second, and third PCs contributed 49.41%, 31.30%, and 16.30% of the total variance, respectively (Table 2). Scores and loading plots of the first two PCs obtained from volatile emissions of six symbionts are presented in Figure 3. The PCA showed three different groupings: *A. murorum* and *Graphium* sp. are located separately in the upper and lower right quadrants, respectively, while *Acremonium* sp., AF-6, AF-8 and *Fusarium* sp. nov. are clustered in the lower left quadrant. Figure 3 displays the variables factor map indicating the discriminating emissions among the symbionts. Compounds **6** (*cis*-*p*-menth-2-en-1-ol), **9** (terpinen-4-ol), **10** (α-terpineol), **11** (*trans*-piperitol), **12** (β-elemene) and **15** (β-bazzanene) are grouped in the lower right associated with *Graphium* sp.; and compounds **1** (α-phellandrene), **2** (α-terpinene), **3** (*p*-cymene), **5** (β-phellandrene), **7** (*cis*-*p*-mentha-2,8-dien-1-ol) and **8** (*trans*-*p*-menth-2-en-1-ol) are grouped in the upper right corner associated with *A. murorum*. In contrast, compounds **4** (limonene), **13** (aristolochene) and **14** (valencene) are grouped in the lower left associated with *Acremonium* sp. and the three *Fusarium* species (AF-6, AF-8 and *Fusarium* sp. nov.). Aristolochene was first isolated from *Aspergillus terreus*, and the headspace profile of *Penicillium roqueforti* and *P. digitatum* cultures were found to be rich in (+)-aristolochene (up to 80%) and valencene (11%) [27].

**Figure 4:**
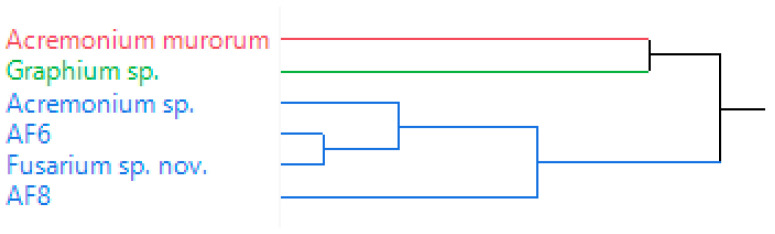
Hierarchical clustering (HCA) was applied using the Ward’s method. Clustering between symbionts and volatile emissions was selected.

A hierarchical cluster analysis (HCA) dendrogram categorized the symbionts into three main clusters (Figure 4). Cluster I and II represented *A. murorum* and *Graphium* sp., respectively. Cluster III grouped *Acremonium* sp., AF-6, AF-8 and *Fusarium* sp. nov, confirming the results from PCA. PCA and HCA results revealed that *cis*-*p*-menth-2-en-1-ol and *trans*-*p*-menth-2-en-1-ol are the key components for discriminating effect of symbionts. This is the first report to compare chemical profiles of the volatile emissions in the six fungal species studied.

### 3.2. cis- and trans-p-Menth-2-en-1-ol Emissions from Graphium sp.

**Figure 5:**
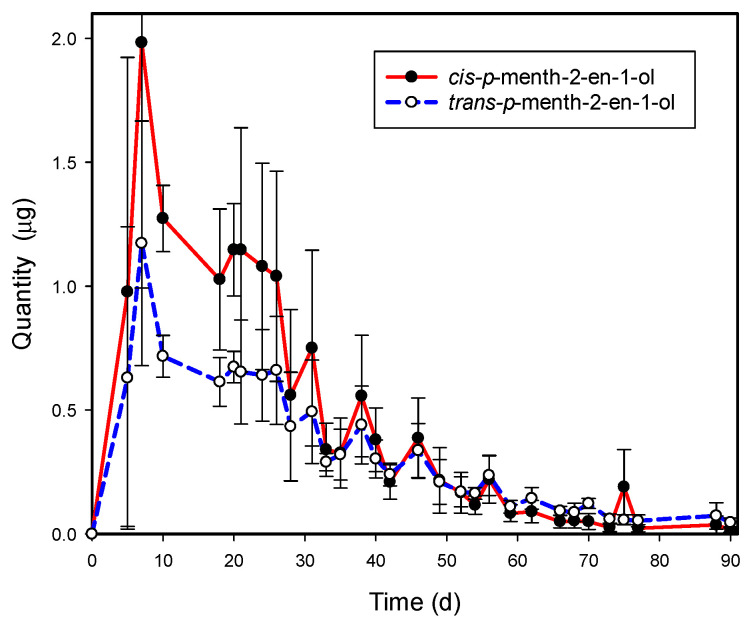
Emissions of *cis*- and *trans*-*p*-menth-2-en-1-ol quantified over time from cultures of *Graphium* sp. Volatiles were isolated by Super-Q collection (15 min adsorption), followed by GC-FID analysis (DB-5 column).

The temporal pattern of emissions for *cis*- and *trans-p*-menth-2-en-1-ol from the *Graphium* sp. cultures is depicted in Figure 5. Based on our experimental design, peak emissions of both compounds were detected at day 7 (although highest levels may have occurred slightly before or after that timepoint since measurements were not recorded daily). Following that peak, emissions began to decline gradually over a period of 90 days. The decrease in emissions over time was best fit by regression with exponential decay models (*cis*: *y* = 2.59*e*^(−0.05*x*)^, *R*^2^ = 0.924; *trans*: *y* = 1.34*e*^(−0.04*x*)^, *R*^2^ = 0.927). Emissions of *cis*-*p*-menth-2-en-1-ol were higher than *trans-p*-menth-2-en-1-ol for the first 30 days, but the difference was significant only on day 10 (*t* = 11.34, df = 2, *p* = 0.004) and day 20 (*t* = 3.24, df = 2, *p* = 0.042).

### 3.3. Laboratory Bioassays

**Figure 6:**
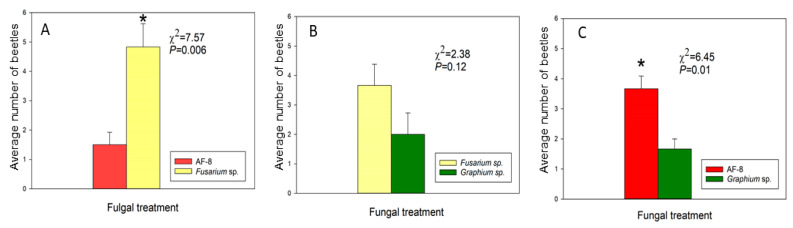
Avocado sawdust disks inoculated with symbiotic fungi and offered to *E. perbrevis* in dual-choice assays. Mean number of *E. perbrevis* in, on or next to the disk after 12 h. (**A**) AF-8 vs. *Fusarium* sp. nov., (**B**) *Fusarium* sp. nov. vs. *Graphium* sp., (**C**) AF-8 vs. *Graphium* sp. * Indicates significant difference in attraction between the two choices (*p* < 0.05).

*Euwallacea perbrevis* females were significantly more attracted to *Fusarium* sp. nov. than to AF-8. When given a choice between AF-8 and *Graphium* sp., females were significantly more attracted to AF-8, but no significant differences were observed when females were offered the *Fusarium* sp. nov. and *Graphium* sp. (*p* = 0.12) (Figure 6).

*trans*-*p*-Menth-2-en-1-ol, detected in the volatiles of all three test fungi, is the main component of the quercivorol lure that is attractive to *E. perbrevis* [13,16] and other *Euwallacea* species [28]. The *cis*-isomer, also present in the quercivorol lure, was only detected in the emissions from *Graphium* sp. *cis-p*-Menth-2-en-1-ol did not increase the attraction of *E. perbrevis* to *Graphium* sp., suggesting that the *trans*-isomer is the primary attractant of *E. perbrevis* and possibly of other *Euwallacea* spp. Furthermore, *trans-p*-menth-2-en-1-ol was the only volatile compound detected in the emissions from all six symbiont cultures (Table 1). Under natural conditions, the fungal gardens of *E. perbrevis* would consist of mixed cultures of all six symbionts, which would emit a strong ‘bouquet’ of *trans-p*-menth-2-en-1-ol.

The two minor components in the commercial lure [16], α- and β-phellandrene, were also detected in the emissions from fungal symbionts. α-Phellandrene was present in emissions from the *Graphium* sp. cultures, and β-phellandrene was emitted by both *Graphium* sp. and AF-8; however, neither compound appeared to increase attraction of female *E. perbrevis* in choice assays. (−)-β-Phellandrene is the predominant monoterpene in the phloem tissue of lodgepole pine (*Pinus contorta* Douglas) and a known kairomone attractant for several species of bark beetle, including the economically important pine engraver, *Ips pini* (Say) [29].

*Graphium* sp. was the least attractive symbiont despite emissions of *trans*-*p*-menth-2-en-1-ol. This suggests that other volatiles from *Graphium* sp. may include potential repellents. For instance, *cis*-*p*-menth-2-en-1-ol and *trans*-piperitol are known to have repellant activity against *Musca domestica* L. and *Haematobia irritans* L. [30,31], and terpinen-4-ol has insecticidal and repellent activities against three stored-product insects: *Tribolium castaneum* Herbst, *Lasioderma serricorne* Fabricius, and *Liposcelis bostrychophila* Badonnel [32]. However, other volatiles produced by *Graphium* sp., like *p*-cymene and β-elemene, are involved in the attraction of *X. glabratus* to Lauraceous trees [33]. More research is needed to understand the effects of these and other volatiles from *Graphium* sp. as potential attractants or repellants of *E. perbrevis*. Identification of effective repellents would facilitate development of a pest management strategy using a ‘push-pull’ approach; a repellent applied to host trees would push pests away, while surrounding traps baited with attractant (i.e., the combination of *trans-p*-menth-2-en-1-ol and α-copaene [5]) would reduce numbers of host-seeking females [34].

*Fusarium* sp. nov. was the most attractive amongst the tested fungi. Besides *trans*-*p*-menth-2-en-1-ol, the only other volatile detected from *Fusarium* sp. nov. was limonene. Therefore, limonene is likely a secondary attractant of *E. perbrevis*, not present in the other two symbionts. Limonene is a known attractant kairomone of another scolytine species, the white pinecone beetle, *Conophthorus coniperda* Schwarz [35]. Limonene is also a precursor in the synthesis of *trans-p*-menth-2-en-1-ol [18]. Given the structural similarities, limonene and *trans-p*-menth-2-en-1-ol may be detected by the same antennal olfactory receptors, but additional electrophysiological studies [5,36] are needed to address that question.

## 4. Conclusions

*Euwallacea perbrevis*, a new invasive ambrosia beetle in Florida, maintains a complex association with multiple species of symbiotic fungi, some of which induce *Fusarium* dieback in host trees, including avocado. This study utilized pure cultures of six symbionts to identify and quantify their volatile emissions, and to determine the attractive components within those emissions. Chemical analyses indicated that *trans-p*-menth-2-en-1-ol was the only compound produced by all six symbionts; behavioral assays indicated that emissions of *cis-p*-menth-2-en-1-ol did not increase attraction. The most attractive symbiont, a new species of *Fusarium*, also emitted limonene, implicating this compound as a secondary attractant. Additional laboratory bioassays and electroantennographic analyses are needed with female *E. perbrevis* to assess limonene for behavioral responses and olfactory chemoreception, respectively. The results presented here may allow improving lures for field monitoring of *E. perbrevis* and possibly other invasive *Euwallacea* species.

## Figures and Tables

**Table 1:** Volatile emissions (mean ± SE) produced by fungal symbionts of *Euwallacea perbrevis* identified by SPME–GC–MS, expressed as the peak area percentages (%).

#	Compounds	* RI Exp	** RI Lit	Composition of Volatile Emissions (Relative % ± SE)					
				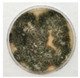	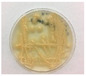	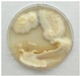	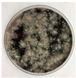	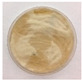	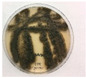
				*Acremonium murorum*	*Acremonium* sp.	AF-6	AF-8	*Fusarium* sp. nov.	*Graphium* sp.
**1**	α-phellandrene ^a^	1007	1002	9.63 ± 2.91	0.00 ± 0.00	0.00 ± 0.00	0.00 ± 0.00	0.00 ± 0.00	4.76 ± 1.33
**2**	α-terpinene ^a^	1018	1014	2.45 ± 0.81	0.00 ± 0.00	0.00 ± 0.00	0.00 ± 0.00	0.00 ± 0.00	2.03 ± 0.69
**3**	*p*-cymene ^a^	1026	1020	1.59 ± 10.67	0.00 ± 0.00	0.00 ± 0.00	0.00 ± 0.00	0.00 ± 0.00	0.87 ± 0.58
**4**	Limonene ^a^	1029	1024	0.00 ± 0.00	31.81 ± 7.66	66.96 ± 3.54	0.00 ± 0.00	80.61 ± 6.76	0.00 ± 0.00
**5**	β-phellandrene ^b^	1030	1025	12.06 ± 3.45	0.00 ± 0.00	0.00 ± 0.00	6.68 ± 1.59	0.00 ± 0.00	7.69 ± 2.13
**6**	*cis*-*p*-menth-2-en-1-ol ^a^	1131	1118	0.71 ± 0.04	20.79 ± 5.25	0.00 ± 0.00	0.00 ± 0.00	0.00 ± 0.00	40.13 ± 4.00
**7**	*cis*-*p*-mentha-2,8-dien-1-ol ^b^	1144	1133	2.16 ± 0.25	0.00 ± 0.00	0.00 ± 0.00	0.00 ± 0.00	0.00 ± 0.00	0.16 ± 0.04
**8**	*trans*-*p*-menth-2-en-1-ol ^a^	1149	1136	71.40 ± 6.02	39.15 ± 6.36	20.38 ± 1.83	23.56 ± 4.36	19.39 ± 6.76	27.32 ± 2.96
**9**	terpinen-4-ol ^a^	1185	1174	0.00 ± 0.00	0.00 ± 0.00	0.00 ± 0.00	0.00 ± 0.00	0.00 ± 0.00	1.92 ± 0.08
**10**	α-terpineol ^a^	1199	1186	0.00 ± 0.00	0.00 ± 0.00	0.00 ± 0.00	0.00 ± 0.00	0.00 ± 0.00	0.68 ± 0.05
**11**	*trans*-piperitol ^b^	1203	1207	0.00 ± 0.00	0.00 ± 0.00	0.00 ± 0.00	0.00 ± 0.00	0.00 ± 0.00	0.32 ± 0.04
**12**	β-elemene ^a^	1394	1389	0.00 ± 0.00	0.00 ± 0.00	0.00 ± 0.00	0.00 ± 0.00	0.00 ± 0.00	0.50 ± 0.11
**13**	Aristolochene ^b^	1486	1496	0.00 ± 0.00	8.25 ± 4.18	12.65 ± 2.00	49.43 ± 3.99	0.00 ± 0.00	10.21 ± 1.59
**14**	Valencene ^a^	1494	1496	0.00 ± 0.00	0.00 ± 0.00	0.00 ± 0.00	20.32 ± 2.33	0.00 ± 0.00	1.14 ± 0.20
**15**	β-bazzanene ^a^	1522	1519	0.00 ± 0.00	0.00 ± 0.00	0.00 ± 0.00	0.00 ± 0.00	0.00 ± 0.00	0.96 ± 0.18
	Total			100.00 ± 0.00	100.00 ± 0.00	99.99 ± 0.00	99.99 ± 0.00	100.00 ± 0.00	98.69 ± 0.20

* RI exp: retention indices calculated on DB-5 column; ** RI lit: retention indices from Adams Library [22]; relative concentration was expressed as the peak area and data listed were the mean of three injection results ± standard error (SE); ^a^ compound identified using retention time of authentic standard, matching with MS libraries and comparison with reported RI; ^b^ compound identified by matching with MS library and comparison with reported RI.

**Table 2:** Loadings, eigenvalues, and percentage of variance for the principal components (PCs) data from symbionts.

Compound	F1	F2	F3	F4	F5
**1**	0.278	0.298	−0.030	−0.138	0.075
**2**	0.333	0.189	−0.029	−0.150	0.070
**3**	0.289	0.280	−0.030	−0.141	0.074
**4**	−0.243	−0.102	−0.418	−0.450	−0.098
**5**	0.261	0.239	0.291	−0.209	−0.254
**6**	0.261	−0.256	−0.079	0.637	−0.169
**7**	0.158	0.415	−0.026	−0.100	0.071
**8**	0.148	0.399	−0.035	0.459	−0.022
**9**	0.307	−0.252	−0.010	−0.101	0.015
**10**	0.307	−0.252	−0.010	−0.101	0.015
**11**	0.307	−0.252	−0.010	−0.101	0.015
**12**	0.307	−0.252	−0.010	−0.101	0.015
**13**	−0.091	−0.115	0.594	−0.013	0.694
**14**	−0.084	−0.048	0.613	−0.121	−0.627
**15**	0.307	−0.252	−0.010	−0.101	0.015
Eigenvalue	7.411	4.694	2.446	0.421	0.027
% Variance	49.407	31.296	16.309	2.809	0.179

## Data Availability

Data are contained within the article.
